# Using an evidence-informed co-design approach to develop a mechanism to measure experiences of the open disclosure process: an Irish case study

**DOI:** 10.1093/intqhc/mzag129

**Published:** 2026-09-07

**Authors:** Aoife De Brún, Eilish McAuliffe, Maria Boylan, Helen Thompson, Catherine Hand, Alexandria Collins, Geraldine Kilkelly, Noreen Kennedy, Clara Meehan, Lorraine Schwanberg, Éidín Ní Shé

**Affiliations:** Centre for Interdisciplinary Research, Education, and Innovation in Health Systems at University College Dublin (UCD IRIS Centre), School of Nursing, Midwifery and Health Systems, University College Dublin, Dublin, Ireland; Centre for Interdisciplinary Research, Education, and Innovation in Health Systems at University College Dublin (UCD IRIS Centre), School of Nursing, Midwifery and Health Systems, University College Dublin, Dublin, Ireland; Healthcare Professional, Dublin, Ireland; Quality and Safety Manager, CHO Dublin North City & County, Health Service Executive, Dublin, Ireland; Incident Management, National Quality and Patient Safety, Health Service Executive, Dublin, Ireland; Advocacy Team Lead, National Patient Advocacy Services, Dublin, Ireland; Quality and Patient Safety Advisor, Galway Roscommon Mental Health Service, Health Service Executive, Galway, Ireland; Quality and Patient Safety Manager, St John’s Hospital, Limerick, Ireland; Patients for Patient Safety Ireland, Ireland; Incident Management, National Quality and Patient Safety, Health Service Executive, Dublin, Ireland; RCSI Graduate School Healthcare Management, Royal College of Surgeons Ireland, Dublin, Ireland; Kemmy Business School, Department of Management and Marketing, University of Limerick, Limerick, Ireland

**Keywords:** co-design, patient experience, open disclosure

## Abstract

**Introduction:**

Open disclosure (OD) is an open discussion with a patient and/or family member about an incident(s) that resulted in harm to that patient while they were receiving healthcare. It is widely considered an ethical and moral imperative and is what patients and families want when harm occurs. However, there is a dearth of research on patient and family experiences of OD and limited measures available to measure the quality of current processes. This research aimed to address this gap by using an evidence-informed approach to working with experts by experience to co-design a mechanism to measure patient experience of OD.

**Methods:**

We adopted a co-design approach, which involves partnering with stakeholders in the healthcare system to address a shared goal and working collaboratively to develop consensus about how best to address an identified challenge. A co-design team was established which included experts by experience (those with direct patient/family experience of OD; *n* = 5), patient advocates (*n* = 2), patient and service user engagement leads in the health system (*n* = 2), quality and patient safety managers/advisors (*n* = 3), members of the National Open Disclosure Team (*n* = 3) and academics/researchers (*n* = 3). A one-day in-person workshop was held using the World Café approach whereby a series of small group discussions on sub-topics helped develop consensus on how to measure experience.

**Results:**

Participants agreed that a brief survey, focusing on key aspects of the OD process would be the optimal way for patients and relevant others to provide feedback that could support learning and quality improvement. Participants acknowledged the use of open text comment boxes within the survey would be useful to allow respondents to elaborate on their experience further, if desired. Furthermore, the survey should provide the option for the respondent to indicate if they would like to speak to someone about their experience of OD in more detail. The co-design team agreed that everyone who has experience of OD, regardless of level of harm, should be eligible and invited to take part in providing feedback on their experiences. A draft survey was developed by the group and is presented in full.

**Conclusions:**

This paper documents the co-design approach adopted on this sensitive topic and shares recommendations that emerged from the co-design process of how experiences of OD should be measured for the purposes of improvement. While the survey was deemed optimal by co-design group members, it is acknowledged that this could be challenging for some to complete. This draft survey, however, offers a valuable starting point to understanding if the process is working as intended for patients and families who experience harm.

## Introduction

Open disclosure (OD) is a process directing healthcare professionals to promptly offer an open and honest apology following harmful patient safety events in care settings to patients and their families and their relevant others through a series of discussions. Open disclosure can be defined as ‘an open discussion with a patient about an incident(s) that resulted in harm to that patient while they were receiving healthcare’ [1]. It is widely considered an ethical and moral imperative, and a growing body of research emphasizes that it is what patients and families want when something goes wrong during the course of healthcare [2, 3]. This process is intended to support patient recovery and to maintain trust in health systems and providers, helping to foster an open and learning culture.

While other terms are used in other jurisdictions, disclosure policies are being increasingly implemented, with the USA, Canada, the UK, New Zealand and Australia considered as early leaders in these efforts [4, 5]. A National Open Disclosure Framework was published in Ireland [6] that described overarching principles to promote a consistent approach to OD in health and social care settings. The first OD policy for the health system was published in 2013 and revised and updated in 2019 [7]. In this policy, it is stated that ‘healthcare staff should communicate with patients and their relevant persons in an open, honest, timely and transparent manner if something goes wrong during care, harm is experienced as a result of care or harm may have occurred as a result of care’ [7]. While the fundamental principles of OD have evolved into an important right for patients who experience harmful events and an obligation on staff involved in those events, the process also plays an integral role in ensuring continuous improvements in the delivery of patient care. When OD does not take place or is unsatisfactory to patients and families, this presents a risk of compounded harm, or the additional harm that may occur if healthcare staff and healthcare organizations are not open and honest and fail to apologize or take responsibility when patient safety incidents occur [8].

Previous research has demonstrated that incident disclosure from health services often does not meet patient and family needs, in terms of the communication with the service, support following the incident and subsequent efforts to prevent recurrence [9]. Recent reviews have highlighted that many of these issues continue to persist across jurisdictions [10]. One issue that is frequently cited is the difference in expectations and attitudes between patients and healthcare professionals regarding disclosure of harm [11] and a gap between ideal disclosure practice and reality [2]. While studies have noted the many barriers and challenges faced by healthcare professionals [11], patient and family needs have been consistently reported on over time. Patients/families want timely, open and honest disclosure conversations, an apology and expression of regret, an explanation for why and how the incident happened and confirmation of tangible changes that will be implemented to avoid incident recurrence [2, 10, 11].

A key mechanism for understanding patient experience of healthcare services is to gather information on the patient experience of service. Patient reported experience measures (PREMs) can offer insight into the quality of a person’s experience of a healthcare service and can direct attention to where improvements or enhancements may be targeted [12]. There is growing evidence for the value of consumer engagement/patient and public involvement in health service monitoring and evaluation [13] and research demonstrates that positive patient experience is associated with higher clinical safety and effectiveness, better health outcomes, and reduced costs [14]. However, there is limited evidence available about the impact of this openness following harm or this expectation of OD [15]. Without PREMs, we are missing a key part of the puzzle in understanding *how* we can improve services and user experiences when harm occurs [12]. A systematic review indicates that the routine collection of patient experience data following engagement in OD processes is remarkably rare [10]. Furthermore, despite increased attention to the topic in recent years, the same issues highlighted in systematic reviews 10–15 years ago are still evident from more recent studies [10]. This underscores the critical importance of gathering patient experience data following individuals’ experience of OD processes and ensuring this informs ongoing efforts to enhance OD processes. It is only by understanding patients and families’ experiences that we can effectively learn and implement changes to processes and to staff training to ensure the process better meets the needs of those who experience harm.

The aim of this research was to collaborate with experts by experience (patients, families and relevant others with experience of the OD process), healthcare staff, patient advocates and researchers to co-design a mechanism to measure patient experience of the OD process.

## Materials and methods

### Research design: co-design

Co-design is critical to designing and delivering services that meet users’ needs (members of the public, patients, families and staff). It involves partnering with stakeholders in the healthcare system to address a shared goal or need and working collaboratively to develop consensus about how best to address an identified challenge. Co-design is a widely used approach for (re-)designing services, implementing quality improvement and ensuring uptake and sustainability of interventions [16, 17]. While there are various approaches to co-design, underpinned by these approaches is a set of values for how people work together to improve service design and delivery [18].

### Identifying relevant stakeholders and recruitment

A one-day co-design workshop took place on 29 April 2024 in Dublin, Ireland. As part of the planning for the workshop with the team in the Health Service Executive (HSE; The HSE in the Irish national health service and manages all public health services in the Republic of Ireland) National Open Disclosure Office, the importance of bringing different experts together was emphasized. Attendees were recruited through health service gatekeepers to ensure a balance between patients, advocates, those in patient support roles and healthcare staff with experience of OD. Recruitment of patients and patient partners was conducted in collaboration with Patients for Patient Safety Ireland (a patient advocacy group with a focus on patient safety) through a gatekeeper who circulated an invitation email on behalf of the research team. In consultation with an advisory group, we aimed to recruit ∼15 people to engage in the co-design workshop. While qualitative co-design work of this nature can never capture all diverse perspectives, we ensured representation from key stakeholder groups, with greatest representation from experts by experience, to ensure their experiences and views shaped this output. Following this outreach, the workshop was attended by 18 people:

1. Experts by experience (those with direct patient/family experience of OD (*n* = 5),
2. Patient advocates (*n* = 2),
3. Patient and Service User Engagement Leads (*n* = 2),
4. Quality and Patient Safety Managers/Advisors and/or OD Leads (*n* = 3),
5. National health service (HSE) Quality and Patient Safety Open Disclosure team members (*n* = 3) with backgrounds in nursing, midwifery, quality and safety and management.
6. Academics/researchers (*n* = 3).

Participants were offered remuneration for their travel costs associated with attending the workshop.

Ethical approval for this research was obtained from the HSE (RRECB0123ADB).

### Co-design approach and workshop structure

**Figure 1:**
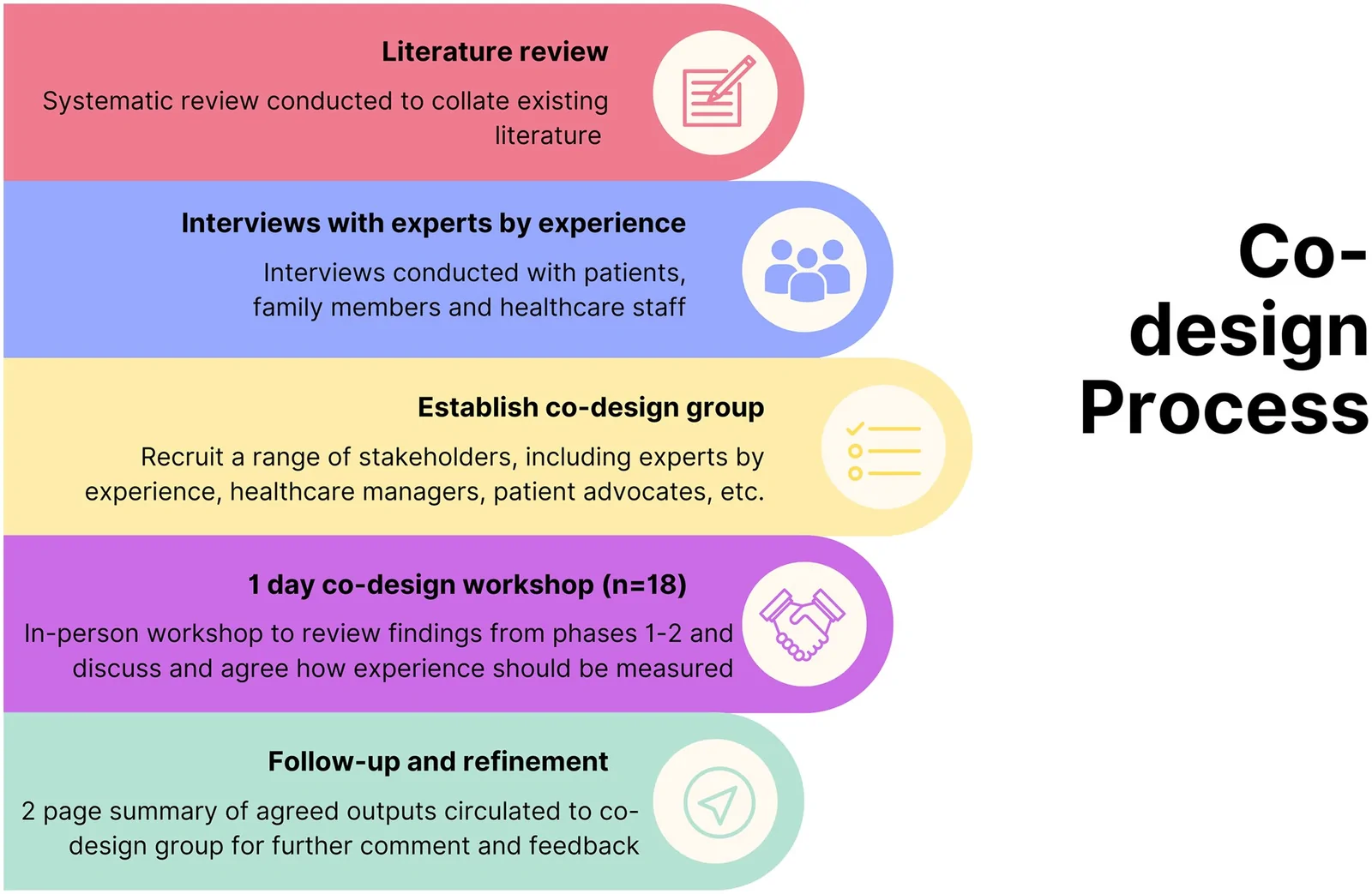
Depiction of steps and inputs of the co-design process.

An evidence-informed co-design approach was adopted [19] that leveraged findings from a systematic review [10] and results of interviews with those with experience with OD in the Irish national context. Figure 1 depicts the inputs and targeting outputs of the process, including the role of stakeholders in the process.

In advance of the workshop, a six-page document was shared with co-design team members advising them of the plan/agenda for the day, some background information on OD and a brief summary of key findings from the interview study that helped inform this work [20]. We also provided examples of how experiences of OD are captured in research in other jurisdictions and advised we would share further examples during the workshop. This pre-briefing helped ensure clarity regarding the purpose and scope of the workshop but also served to share information to help participants prepare to fully engage with the workshop.

**Figure 2:**
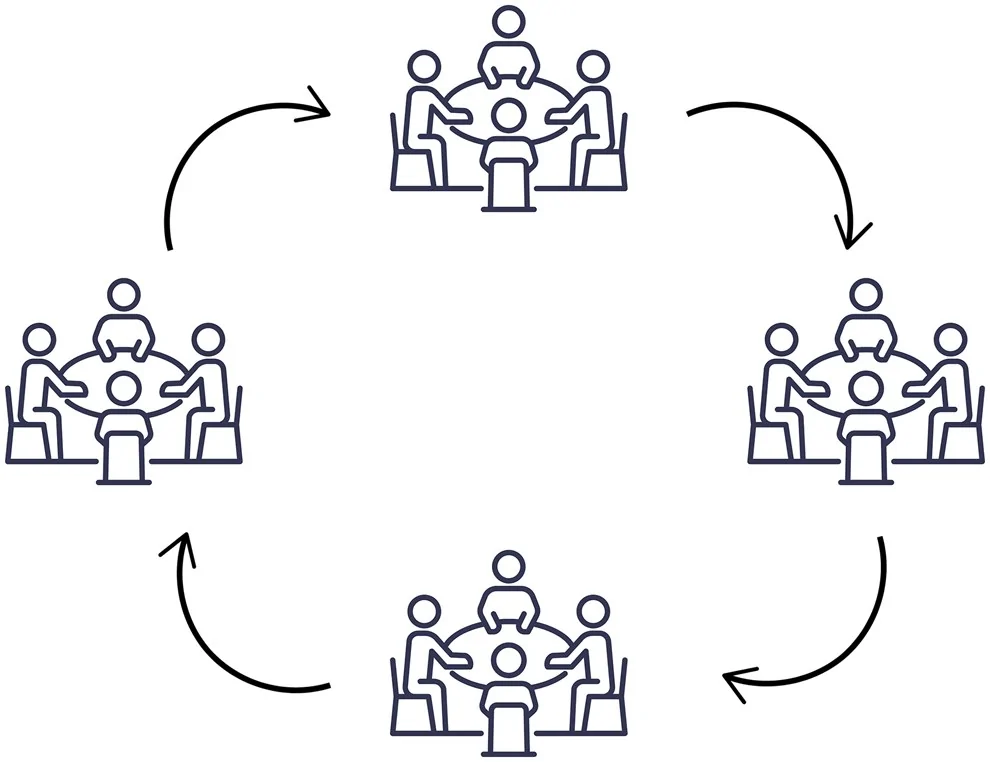
World Café process and round questions. *Round 1*: what’s important to patients? From your perspective, on this topic, what should we focus on? What does the evidence from the research suggest we should focus on? *Round 2*: review the notes made by the previous group. What are the types of questions we should ask to understand this aspect of experience? How should we phrase these items/questions so that they are clear but also sensitive to respondents? *Round 3*: review and build on questions/items developed and selected by other groups. Continue to refine as needed. *Round 4*: review notes from round 1 and progress and changes made by other groups. Is anything missing that could be important to add on this topic?.

During the workshop, we employed the World Café format [21] to support an inclusive approach to ensure all attendees could contribute their thoughts and ideas to discussions on various aspects of the OD process. The World Café methodology is a simple and flexible format for hosting large group discussions [21]. The room was set up with four tables, and four discussion rounds of 20 minutes each took place, where after each round, participants moved to another table to discuss another topic, building on the discussion and ideas of the group that went before them (Fig. 2). Each table had a host to guide and facilitate the discussion on the topic or question. For the purposes of this discussion, each table had a group of similar topics or aspects of the OD process. There were four discussion tables and each had its own topic that was covered: one focused on initial disclosure (including timeliness, apology and acknowledgement); one on communication (including clarity, understanding and quality); another discussed openness, transparency, respect and dignity and the final table covered next steps, continued communication and efforts to avoid recurrence of incident. This was based on results from an earlier phase of this work (summarized in Table 1) and on evidence from the literature. During discussions, participants could add topics they felt were important for consideration. Previous research [10] and scales [22–24] and measurement approaches used in other settings were presented and considered by the group.

**Table 1:** Summary of key findings from qualitative work to inform co-design process (adapted from Ref. [20]).

Topic		Sample quotes
Importance of acknowledgement and apology	Participants recognized the need to have an explicit acknowledgement of what had happened to them, but some patients felt they did not have this.	‘There was never really a kind of an actual acknowledgement at that time that oh that shouldn’t have happened. Or you know it was more just this kind of gloss over, “oh we understand your concern,” or “we’ll take that onboard.”’ (Family member) ‘The whole process for me was a fight and a battle. But in the end, I got my apology, so I did, I asked for my apology. That’s all I wanted, an acknowledgement and an apology.’ (Patient)
Clarity of communication regarding open disclosure	Reluctance among staff to use the term ‘open disclosure’ and as a result, there was a lack of clarity for patients and families.	‘I suppose my biggest concern and issue in open disclosure is that the words open disclosure are rarely used….[] So sometimes it’s actually very unclear whether the person has actually had an open disclosure meeting or not.’ (Patient Advocate)
Staff being prepared for open disclosure	Participants reported frustration at feeling staff were ill-informed or unprepared for open disclosure meetings.	‘The consultant knew there was a need for an open disclosure meeting and I felt a bit uneasy going into the meeting without having all the evidence before me.’ (Healthcare professional)
Openness and transparency	Participants expected openness and transparency and described the negative impact on healing and trust when not evident.	‘I actually appreciated, if I’m honest, I remember back at the time, I appreciated their honesty. I was a bit annoyed, but I appreciated their honesty.’ (Patient) ‘[If] you are missing a few pieces [of information about what happened] and then somebody says no you can’t have it. And it just makes things much worse.’ (Patient)
Need for space to share story	Both patients and healthcare professionals recognized the importance of providing those impacted by a patient safety incident space to share their story and experiences.	‘That they get to tell their story, [it’s a] basic human right that they’re heard and understood… really you have to let people tell their story…. that’s the key to that connection.’ (Healthcare professional)
Feeling incident was dismissed or minimized	Some participants reported poor experiences of communication with healthcare professionals whom they felt were dismissing or minimizing the impact of the incident on them.	‘They just really played down the severity of the whole thing.’ (Patient)

Through the World Café method and having various timed rounds of discussion, with smaller groups moving around tables, each new group could add to, amend and revise the work of the previous group, enduring refinement and synthesis were constantly occurring during the workshop. At the end of the workshop, the outputs from each table were presented back to the full group for a final round of feedback. This also enabled a sense check and ensured that content represented the views of all who had contributed. The final output and recommendations were agreed by the group.

After the workshop, a written summary of this agreed output was compiled, and there was further opportunity for co-design team members to reflect and provide feedback. Team members could provide further amendments or additions before the document was agreed and finalized by the group (reported herein).

## Results

### Recommended approach for measuring experience of OD

Co-design team members discussed the various options for collection of data on experiences, including individual interviews and a survey to gather experiences. Following discussion regarding the advantages and limitations of these options, participants agreed that a brief survey, focusing on key aspects of the OD process would be the optimal way for patients and relevant others to provide feedback that could support learning and quality improvement. It was felt that the survey approach would facilitate anonymity, which was deemed crucial for the receipt of honest feedback, without fear of recrimination, as fear of reprisals is known to be a barrier to providing feedback on safety and care concerns [25].

However, participants noted that some respondents may prefer to tell someone about the totality of their experience and that this should be an option for those that would like to feedback in this way. Therefore, it was suggested that the survey should include an open text comment box for individuals to elaborate on their experience if they would like to. Furthermore, the survey should provide the option for the respondent to indicate if they would like to speak to someone about their experience of OD in more detail (e.g. an interview with a member of the OD/patient experience team). To manage expectations, it should be clearly communicated that the purpose of this interview would be to gather feedback to improve the OD experience for future patients and family members. Co-design team members felt that this person-centred approach would put the power and choice in the hands of people who have experienced harm and allow them to provide feedback in a manner that suited them. Furthermore, given the sensitive nature of the discussion, it would be appropriate for those gathering this feedback from patients through interview to have advanced skills and training in qualitative interviewing on sensitive topics. It would also be important to have a clear protocol for managing distress, if the person became distressed during this discussion [26–28].

### Eligibility for and timing of participation

The co-design team agreed that everyone who has experience of OD, regardless of level of harm or whether OD happens ‘at the bedside’ or in a meeting room, should be eligible and invited to take part in providing feedback on their experiences. There was consensus that there should be no time indications or limitations on when people can provide this feedback following their involvement in OD. The group felt that there is no way to determine an ‘appropriate’ period following engagement in the OD process, regardless of the level of harm experienced. Instead, the group believed that this decision should reside with the person. The group agreed that this person-centred approach to gathering feedback would be the most sensitive and appropriate, given that every patient safety incident is unique and would put the choice and power in the patient/relevant other’s hands, giving them the information and allowing them the choice of when and how to give feedback.

International evidence demonstrates that not all harmful patient safety incidents are reported [2], and that OD does not always occur following harmful patient safety incidents [29, 30]. Given this, there were concerns expressed by group members that only inviting feedback from those who have had their details captured in the national incident management systems might exclude some people with relevant experience of the process. There was discussion about imminent changes to the national systems to normalize the capture of OD cases, but this is not currently in place. Instead, participants suggested the most appropriate means to create awareness of feedback mechanisms was through promoting the survey (using QR codes and links) on OD information leaflets, in OD packs in organizations, on posters in healthcare settings, on health service websites, via staff newsletters, patient partner organization websites/social media, professional and regulatory bodies. The group were aware that surveys are not necessarily accessible to everyone and that additional supports would likely be required to be offered for those who, due to, for instance, language or literacy barriers, may find completion of a survey challenging. In sum, the group did not feel a targeted invitation approach was appropriate given the sensitivities inherent in cases requiring OD.

### Co-design output: draft survey

Through a series of iterative discussions, the co-design team considered the evidence presented by the research team on the findings from the literature and from interviews with those with experience of OD in the Irish context. Taken together, the group prioritized a series of questions to address important aspects of the process. This survey was reviewed again by co-design team members following the workshop for any further suggestions and amendments. Furthermore, many of the co-design participants are co-authors of this paper, underlining the central role they have played in the development of this work. The survey addresses key aspects of the OD process including the timeliness of the disclosure, perceived sincerity of the apology, whether the person feels learning has occurred because of the incident and several questions regarding the clarity and quality of communication. The full survey is available in Appendix 1.

## Discussion

### Statement of principal findings

This paper has detailed the process, inputs and resulting proposed mechanisms for the measurement of patient and their relevant other(s) experience of the OD process. Through an evidence-informed approach and partnership with relevant experts by experience, a survey that addresses key aspects of the OD process has been co-designed to capture experiences of specific aspects of the OD process, including questions addressing the timeliness of disclosure, whether an apology was offered, and the perceived quality and clarity of communication.

### Strengths and limitations

A key strength of this work was the evidence-informed, co-design approach adopted to designing a patient experience measure of OD that was sensitive, appropriate and that addressed key aspects of the OD process where experience is shaped. Casaca *et al.* [12] describe the ‘essential role of co-development and stakeholder engagement’ (p. 1) in developing PREMs and patient-reported outcome measures, and the importance of balancing rigour with practicality around data collection and acceptability and data collection efforts. By leveraging the available evidence and harnessing the experiences of those who have been through the process, the co-design group developed a survey to obtain feedback to understand if various aspects of the process are working for patients or not.

While this study has commenced the design of a tool, further cross-cultural pilot testing would be required to evaluate the usability and understanding of the survey in advance of implementation. It is recommended that a cognitive interviewing approach [31] be adopted to ensure the questions are being understood as intended and to clarify language, where appropriate. It would be also important to ensure that the survey and feedback mechanisms support those who may require support or translation services to provide feedback on their experiences.

### Interpretation within context of wider literature

International evidence demonstrates that OD does not happen in all harmful patient safety incidents [5]. In the Irish national context, there is not yet a clear sense of how often OD happens in practice, and if the OD process in its current iteration is meeting the needs of patients, families and their relevant others. This research has leveraged existing research on patient experiences of OD to inform and guide the co-design of a mechanism to capture feedback on experiences of OD for the purposes of learning and improvement. Ongoing monitoring and feedback mechanisms are needed to continuously improve and bring greater quality and consistency to the practice. National measurement data on experiences of OD are crucial to understand experiences of the process and to inform targets for improvement.

A review of the literature has pointed to findings that relatively few studies have measured the actual experiences of patients and families with the disclosure process, and where it is measured, this has been measured in various ways, with no consistency in use of scale or questions [10]. Given that processes and procedures regarding OD differ across jurisdictions, this is understandable. However, there are likely to be questions that represent aspects that are core or shared across processes, as well as those that are more tailored to specific contexts and their processes. Through literature review to harness international research [10] and interviews to understand local experiences [20] we have identified the aspects of the process that shape family and patient experience and have worked with experts by experience to develop a sensitive and appropriate way to do this. The co-design survey addresses the consistent concerns that have been reported in the literature, including the importance of an apology, a timely disclosure, clarity and sensitivity in communication, affording an opportunity to share the impact of the incident, the availability of necessary emotional support and confidence that learning would occur in the service as a result of the incident.

According to Wu *et al.* [5], ‘common metrics need to be developed to allow monitoring and evaluation of performance, identification of best practices and opportunities for quality improvement, and accountability to regulatory bodies and the public’ (p. 5). We hope that the current work will provide a catalyst for research on the measurement of patient experience to understand how we can move towards these common approaches to measurement to learn from local and international experiences.

### Implications for policy, practice and research

Health systems can learn from patients, families and the public regarding how to improve the quality and safety of care. There is a strong case for patients and families being an additional safety ‘scaffold’ in the system and source of insight in the system to promote and support safety [32]. Indeed studies have demonstrated that patients can identify when they have experienced harm, with positive associations recorded between medical documentation analysis and patients’ reporting of their own experience [33]. This demonstrates that patients can and do provide valuable feedback to improve systems, and it is incumbent on healthcare systems to seek out and act on feedback received to ensure quality care that meets patient needs [12].

Prior to implementation, this survey should be pilot tested to ensure that the questions are interpreted and understood as intended. We encourage researchers to move towards a ‘core’ set of questions, so that we can begin to share learning internationally from the results of experience surveys to understand patient and family experiences of disclosure processes across health systems.

## Conclusions

Through an evidence-informed approach and partnership with experts by experience, a survey that addresses key aspects of the OD process has been co-designed. This survey addresses key points where experience is shaped, including the perceived timeliness of the disclosure, the offering of a sincere apology, listening to the person’s experience and whether it is perceived that learning has occurred as a result of the incident.

## Data Availability

Given the sensitive nature of the data, data are not publicly available but are available from the corresponding author (A.D.B.) upon reasonable request.

## Appendix 1

**Draft Survey** 

**Open disclosure: Patient/relevant other survey of experiences** 

You have been invited to take part in this survey as you have recently had an experience of open disclosure, where staff told you about something that went wrong during the course of your or your loved one’s healthcare. We want to learn from your experience of this discussion with the healthcare team to know how we can improve this experience for people in the future. By sharing your experience of what went well and what perhaps did not go well, we hope to inform how we train and support staff.

Was the term ‘open disclosure’ used by staff and explained to you?

☐ Yes☐ To some extent/somewhat☐ No

Were you aware of the purpose of the open disclosure meeting?

☐ Yes☐ To some extent/somewhat☐ No

Were you informed that you could have a support person/independent advocate at the open disclosure meeting with you?

☐ Yes☐ To some extent/somewhat☐ No

Did the disclosure of what went wrong take place in a timely manner (at the earliest opportunity)?

☐ Yes☐ To some extent/somewhat☐ No

Did you feel the apology was sincere?

☐ Yes☐ To some extent/somewhat☐ No

Were you treated with respect, empathy, and compassion?

☐ Yes☐ To some extent/somewhat☐ No

Did the staff and organisation acknowledge that a patient safety incident had occurred that impacted you?

☐ Yes☐ To some extent/somewhat☐ No

Was enough time given for this open disclosure meeting?

☐ Yes☐ To some extent/somewhat☐ No

Were you assigned a contact person to support your communication regarding this incident?

☐ Yes☐ To some extent/somewhat☐ No

Did you feel that the staff were adequately prepared for the meeting?

☐ Yes☐ To some extent/somewhat☐ No

Did you feel staff had the knowledge to answer your questions?

☐ Yes☐ To some extent/somewhat☐ No

Did you feel comfortable asking questions and expressing concerns to the healthcare team?

☐ Yes☐ To some extent/somewhat☐ No

Were you given meaningful, truthful, and clear answers and information in response to all your queries and concerns?

☐ Yes☐ To some extent/somewhat☐ No

Where your expectations were not met or you were not satisfied, were you given clear explanations for why this was not possible?

☐ Yes☐ To some extent/somewhat☐ No

Were you assured that information you were discussing would be kept confidential?

☐ Yes☐ To some extent/somewhat☐ No

Was the language used by healthcare staff clear and easy to understand?

☐ Yes☐ To some extent/somewhat☐ No

Did you have an opportunity to share your personal experience of what happened and its impact on you with the healthcare team?

☐ Yes☐ To some extent/somewhat☐ No

Were you offered or provided with information including supports available to you, including independent patient advocate services or counselling supports?

☐ Yes☐ To some extent/somewhat☐ No

Were you asked for your suggestions to reduce the risk of this happening to someone else?

☐ Yes☐ To some extent/somewhat☐ No

Were you confident that learning occurred and that actions were taken to try to prevent the same thing happening to other patients?

☐ Yes☐ To some extent/somewhat☐ No

Were you offered a follow-up meeting?

☐ Yes☐ Not sure☐ No

Please share any additional comments in relation to your experience of the open disclosure process (positive or negative).

If you would like to speak to someone about your experience of open disclosure in more detail, you can indicate this below and include your contact details. By doing this, you are agreeing for a member of the team to contact you for the purpose of arranging a call/meeting to discuss your experience in more detail to understand what we can learn to try to improve people’s experience in the future. This is optional. Please only complete this section if you are happy to be contacted and to discuss your experience further.

Name: Contact details:

Thank you for sharing your feedback. This information will be compiled with the responses of others to inform how we can improve open disclosure and make sure it meets the needs of our patients, service users and their families.

*END*
